# Association of Alkaline Phosphatase with Cardiovascular Disease in Patients with Dyslipidemia: A 6-Year Retrospective Study

**DOI:** 10.3390/jcdd11020060

**Published:** 2024-02-15

**Authors:** Petros Spyridonas Adamidis, Matilda Florentin, Evangelos Liberopoulos, Amalia Despoina Koutsogianni, Georgia Anastasiou, George Liamis, Haralampos Milionis, Fotios Barkas

**Affiliations:** 1 Department of Internal Medicine, Faculty of Medicine, School of Health Sciences, University of Ioannina, 45110 Ioannina, Greece; padam7@yahoo.gr (P.S.A.); matildaflorentin@yahoo.com (M.F.); amaliadespoina.koutsogianni@gmail.com (A.D.K.); anastgeorgia@hotmail.com (G.A.); gliamis@uoi.gr (G.L.); hmilioni@uoi.gr (H.M.); 2 1st Propedeutic Department of Medicine, School of Medicine, Laiko General Hospital, National and Kapodistrian University of Athens, 15772 Athens, Greece; elibero@med.uoa.gr

**Keywords:** alkaline phosphatase, cardiovascular disease, cholesterol, statins

## Abstract

Background and Aim: Serum alkaline phosphatase (ALP) activity has been associated with atherosclerotic cardiovascular disease (ASCVD). We aimed to investigate the association of ALP with ASCVD in patients with dyslipidemia. Methods: We conducted a retrospective cohort study including consecutive adults with dyslipidemia followed-up for ≥3 years (from 1999 to 2022) in the outpatient Lipid Clinic of Ioannina University General Hospital, Greece. The primary endpoint was the association between baseline ALP and incident ASCVD after adjusting for traditional risk factors (i.e., sex, age, hypertension, diabetes, smoking, and dyslipidemia), baseline ASCVD, and lipid-lowering treatment. ALP levels were stratified by tertiles as follows: low: <67 U/L, middle: 67–79 U/L, high: ≥79 U/L. Results: Overall, 1178 subjects were included; 44% were males, and their median age was 57 years (range: 49–65). During a 6-year median follow-up (interquartile range: IQR: 4–9), 78 new ASCVD events (6.6%) occurred. A statistically significant association between baseline ALP levels and incident ASCVD was demonstrated (Odds Ratio, OR: 6.99; 95% Confidence Interval, CI: 2.29–21.03, *p* = 0.001). Subjects in the highest ALP tertile had the highest odds for ASCVD when compared with those in the lowest tertile (OR: 2.35; 95% CI: 1.24–4.41, *p* = 0.008). Conclusions: The present study indicates an association between ALP and the development of ASCVD in patients with dyslipidemia, which underscores the potential of ALP as a predictive tool or a therapeutic target in the realm of ASCVD prevention within this population.

## 1. Introduction

Atherosclerotic cardiovascular disease (ASCVD) stands out as a leading cause of global mortality, underscoring the critical role of modifiable risk factors in its occurrence and associated mortality [1]. Among these factors, elevated levels of low-density lipoprotein cholesterol (LDL-C) have been identified as a major contributor to ASCVD [2]. The proven benefits of LDL-C reduction through existing lipid-lowering regimens have significantly impacted both primary and secondary prevention efforts [2]. Despite the successes of current treatments, a noteworthy proportion of optimally managed patients continue to face the risk of cardiovascular events and mortality, suggesting the existence of residual cardiovascular risk [3]. The emerging evidence from retrospective and prospective studies, encompassing populations with and without established cardiovascular disease (CVD), indicates that baseline serum alkaline phosphatase (ALP) levels might serve as a valuable predictive marker for overall mortality and cardiovascular risk [4,5,6,7,8]. It is particularly intriguing that recent research has explored the potential of drug-induced reduction in ALP to mitigate cardiovascular risk in individuals with ASCVD [9]. A notable example is a phase 2 placebo-controlled trial involving apabetalone, conducted with 795 patients already diagnosed with ASCVD and undergoing statin therapy. This trial demonstrated a significant reduction in major adverse cardiovascular events (MACEs) when ALP levels were lowered by 1 standard deviation (1-SD) after 24–26 weeks of treatment, with a hazard ratio (HR) of 0.64 (95% confidence interval, CI: 0.46–0.90, *p* = 0.009) [9].

Against this backdrop, our study aims to investigate the potential role of ALP as a predictive biomarker for ASCVD and as a promising therapeutic target in patients with dyslipidemia. Our investigation specifically involves scrutinizing the association between baseline ALP levels and the incidence of ASCVD while meticulously adjusting for traditional risk factors such as sex, age, hypertension, diabetes, smoking, and dyslipidemia, as well as baseline ASCVD and ongoing lipid-lowering treatments. This comprehensive approach seeks to unravel the intricate interplay between ALP levels and cardiovascular outcomes, offering insights that could potentially refine risk stratification and inform targeted therapeutic interventions in the realm of cardiovascular health.

## 2. Materials and Methods

This retrospective cohort study involved the comprehensive analysis of 1178 Caucasian patients of Hellenic origin diagnosed with dyslipidemia. The participants were enrolled in the Outpatient Lipid Clinic of the University Hospital of Ioannina, Greece, and had a minimum follow-up duration of three years, spanning the period between 1999 and 2022. The study protocol obtained ethical approval from the local Institutional Ethics Committee, ensuring compliance with established ethical standards. Additionally, informed consent was diligently obtained from all participating patients.

Detailed cardiovascular histories and relevant medication profiles were thoroughly documented for each patient. A detailed documentation of patient demographic and clinical characteristics was conducted, focusing on sex, age, smoking, and concomitant diseases, with a specific emphasis on ASCVD and cardiovascular risk factors such as age, smoking status, hypertension, diabetes, and chronic kidney disease.

Clinical and laboratory data were meticulously gathered to unravel the intricate relationships between cardiovascular health, laboratory parameters, and clinical attributes. Blood pressure measurements adhered to the European Society of Cardiology (ESC)/European Society of Hypertension (ESH) guidelines, utilizing a validated upper-arm cuff BP measurement device and an appropriate cuff size [10]. Blood serum samples were collected in the morning into sterile Vacutainer-SST II advance tubes (Becton-Dickinson, Plymouth, UK) after overnight fasting for at least 8–12 h. Tubes were refrigerated immediately after collection, centrifuged at 4 °C within 40 min of blood sampling, and then analyzed within 2 h. A comprehensive lipid profile, including total cholesterol, triglycerides, high-density lipoprotein cholesterol (HDL-C), and LDL-C, underwent precise analysis. Serum concentrations of total cholesterol were determined enzymatically on an Olympus AU600 Clinical Chemistry Analyzer (Olympus Diagnostica, Hamburg, Germany). HDL-C was determined by a direct assay (Olympus Diagnostica, Hamburg, Germany). LDL-C was calculated using the Friedwald formula, provided that triglycerides were <400 mg/dL (4.5 mmol/L). Furthermore, a complete blood count, creatinine, urea, electrolytes, liver enzymes, creatinine kinase, thyroid function, and urine analysis were assessed. The renal function was estimated using the estimated Glomerular Filtration Rate (eGFR) calculated with the CKD-EPI (CKD Epidemiology Collaboration) formula, utilizing creatinine results calibrated to isotope dilution mass spectrometry. All samples were measured using the same methods and analyzers under similar conditions during the follow-up of this study at the Biochemistry Department of the University General Hospital of Ioannina, Ioannina, Greece.

Cardiovascular and metabolic conditions were comprehensively defined, encompassing a spectrum of ASCVD, including Coronary Artery Disease (CAD; myocardial infarction, stable angina, unstable angina, percutaneous coronary intervention, coronary artery bypass graft surgery), stroke (ischemic stroke, hemorrhagic stroke, and transient ischemic attack), Peripheral Artery Disease (PAD; history of claudication plus ankle-brachial index <0.9, or previous revascularization or amputation), and carotid stenosis >50%. Precise diagnostic criteria were established for hypertension, diabetes, and impaired fasting glucose, adhering to ESC/ESH and ESC/European Association for the Study of Diabetes (EASD) guidelines [11]. The diagnosis of chronic kidney disease (CKD) was contingent upon observing a decline in eGFR <60 mL/min/1.73 m² in two periodical examinations over at least a 3-month timeline.

Concomitant therapy, with a particular focus on lipid-lowering drugs such as statins, ezetimibe, proprotein convertase subtilisin/kexin type 9 inhibitors, fibrates, colesevelam, and omega-3 fatty acids, was diligently recorded. The intensity of statin therapy was classified as ‘high,’ ‘moderate,’ and ‘low’ based on the anticipated average LDL-C lowering of ≥50, 30–50, and <30%, respectively.

ALP levels were categorized into tertiles (‘low’ < 67 U/L, ‘middle’ 67–79 U/L, and ‘high’ ≥ 79 U/L), offering insights into ALP concentrations within the study population. This stratification aimed to facilitate a comprehensive analysis of the potential association between ALP and cardiovascular outcomes.

### Statistical Analysis

Continuous variables underwent normality testing using the Kolmogorov–Smirnov test, and logarithmic transformations were applied if necessary. Parametric data are presented as mean ± standard deviation (SD), while non-parametric data are expressed as median (interquartile range (IQR)). Categorical values are presented as frequency counts and percentages. Paired sample t-tests, both parametric and non-parametric, were employed to examine changes in numeric variables during the follow-up period within each group.

Pearson’s and Spearman’s correlation coefficients were utilized to explore the relationship between ALP changes and other variables. A multivariate logistic regression analysis was conducted to investigate the impact of ALP changes on the development of ASCVD, adjusting for potential confounding factors. Associations with ASCVD outcomes are reported as odds ratios (OR) with an accompanying 95% CI. Significance was set at *p* < 0.05, and all analyses were conducted using the Statistical Package for Social Sciences (SPSS) v21.0 software (SPSS IBM Corporation, Armonk, New York, NY, USA). The rigorous methodology employed in this study aimed to provide robust insights into the intricate relationships between ALP, dyslipidemia, and ASCVD incidence within this specific cohort.

## 3. Results

A total of 1178 subjects with dyslipidemia were enrolled in this study. Among the participants, 44% were male, with a median age of 57 years (range, 49–65). Notably, 32% of the cohort were smokers, and the prevalence of hypertension, diabetes, and chronic kidney disease was observed in 61%, 11%, and 10% of the subjects, respectively. Furthermore, 16% of the total sample had established ASCVD, and 271 individuals were on statin therapy at the initiation visit. The majority of those were on statin therapy of moderate-intensity, and only a small proportion were treated with non-statin lipid-lowering drugs (Table 1).

A detailed examination of baseline characteristics, as presented in Table 1, revealed significant insights. Significant variations were noted among the three groups of subjects stratified by ALP tertiles, particularly regarding traditional cardiovascular risk factors and the usage of lipid-lowering therapy. The highest ALP tertile group demonstrated a reduced percentage of smokers and lower HDL-C levels compared to other participants (*p* < 0.001). However, a greater proportion of those with the highest ALP levels were older and exhibited higher levels of blood pressure, fasting plasma glucose, total cholesterol, triglycerides, and LDL-C, according to the lower rates of lipid-lowering therapy (*p* < 0.05 for all comparisons across ALP tertile groups). This trend was more evident in the rates of statin treatment, while no major differences were noted regarding the statin potency or the rates of non-statin lipid-lowering drugs (Table 1). Laboratory variables also demonstrated differences (Table S1), with higher mean values of calcium and direct bilirubin found at the middle and lower tertiles of ALP, respectively (*p* < 0.05).

Diversities were also noted across study participants when stratified by lipid-lowering therapy; those taking lipid-lowering drugs had lower levels of alkaline phosphatase, blood pressure, total cholesterol, HDL-C, LDL-C, and higher fasting plasma glucose levels when compared with those not taking lipid-lowering drugs at the baseline visit (Table S2).

In a fully adjusted model accounting for age, sex, hypertension, diabetes, smoking, baseline ASCVD, triglycerides, HDL-C, LDL-C, and lipid-lowering therapy, the ALP levels demonstrated a significant association with higher incident ASCVD (OR: 6.99; 95% CI: 2.29–21.03, *p* = 0.001; Table 2). This finding remained significant regardless of lipid-lowering therapy (OR: 4.74; 95% CI: 1.37–16.41, *p* = 0.014 for the participants not taking lipid-lowering drugs; OR: 28.63; 95% CI: 2.19–373.71, *p* = 0.010 for the participants on lipid-lowering therapy). 

When subjects were stratified by ALP tertiles, the highest tertile group exhibited a higher odd for incident ASCVD in the fully adjusted model (OR: 2.35; 95% CI: 1.24–4.41, *p* = 0.008). Consistent results were obtained in less-adjusted analytical models, both in a crude analysis and an adjusted model (Models 1 and 2, Table 2). The association between ALP levels as a continuous variable and incident ASCVD was significant in both models (OR: 8.67; 95% CI: 3.21–23.38, *p* < 0.001 and OR: 9.10; 95% CI: 3.22–25.75, *p* < 0.001, respectively). Furthermore, a similar significant association was observed for the highest tertile of ALP within both analytical models (OR: 2.79; 95% CI: 1.54–5.04, *p* = 0.001 and OR: 2.72; 95% CI: 1.49–4.98, *p* = 0.001, respectively).

## 4. Discussion

The present study demonstrated that ALP is associated with ASCVD development in patients with dyslipidemia. These robust associations, consistently observed across various analytical models, underscore the potential predictive role of ALP levels, particularly in its highest tertile, in incident ASCVD. Our fully adjusted model, considering an array of demographic and clinical factors, further emphasizes the strength of this association, providing valuable insights into the link between ALP and cardiovascular outcomes in individuals with dyslipidemia. 

Our study’s prospective design aligns with existing research, particularly a comprehensive 10-year cohort involving 6974 subjects from the general population [8]. This seminal investigation revealed a compelling and independent association between serum ALP activity and ASCVD, underscoring the significance of ALP as a potential cardiovascular risk marker [8]. The HR of 1.34 (95% CI: 1.14–1.56, *p* < 0.001) indicated a substantial increase in ASCVD risk associated with elevated ALP levels [8]. The interconnectedness of ALP and C-reactive protein (CRP) was further illuminated in a study involving 4155 subjects over 20 years old, hinting at a shared biological pathway between these two parameters [12,13,14]. Expanding the scope to a prospective study involving 3381 men aged 60 to 79 years, the association between elevated ALP levels and adverse cardiovascular outcomes became more pronounced [15]. The heightened risk of CAD (HR: 1.90; 95% CI: 1.40–2.56, *p* < 0.0001), CVD mortality (HR: 1.72; 95% CI: 1.27–2.32, *p* < 0.0001), and overall CVD events (HR: 1.73; 95% CI: 1.39–2.16, *p* < 0.0001) in elderly men with elevated ALP levels reinforces the potential utility of ALP as a prognostic marker in this demographic [15]. Crucially, this association retained significance even after adjustment for a spectrum of cardiovascular risk factors and CRP levels, with an adjusted HR of 1.19 (95% CI: 1.05–1.34, *p* = 0.007) [15]. This adjustment accounts for confounding variables and affirms the robustness of the association [15]. Furthermore, the broader landscape of observational studies strengthens the case for ALP as a predictor of cardiovascular outcomes [5,6]. A systematic review and meta-analysis of 24 observational studies, encompassing 147,634 patients, provided compelling evidence for a positive association between serum ALP activity and total mortality in individuals with normal renal function [5]. The pooled risk ratio (RR) of 1.57 (95% CI: 1.27–1.95) suggests a significant correlation between elevated ALP levels and increased mortality risk in this population [5]. A meta-analysis of four prospective studies, involving 33,727 participants, contributed further weight to the association between ALP and ASCVD [4]. Reporting an 8% (95% CI: 3–14%) higher risk of ASCVD per 1 standard deviation increase in baseline ALP activity, this meta-analysis underscores the consistency of findings across diverse study populations and settings [4]. Therefore, the wealth of evidence presented across various studies, including our own, emphasizes the importance of ALP as a potential biomarker for ASCVD risk assessment. The exploration of the association of the ALP with cardiovascular outcomes, especially in the context of inflammation and aging, enriches our understanding of its clinical relevance. Future research should delve deeper into the mechanistic underpinnings of this association and explore the potential of ALP as a target for interventions aimed at reducing cardiovascular risk.

The intricate pathophysiology associated with alkaline phosphatase (ALP) extends beyond a mere biomarker, revealing its multifaceted involvement in cardiovascular health. Conditions characterized by heightened oxidative stress, such as CKD, have been identified as contributors to increased ALP activity [16]. This elevation in ALP activity is implicated in various deleterious processes that collectively impact cardiovascular health [16]. One notable consequence of enhanced ALP activity is the potential induction of vascular calcification, a pathological process associated with the deposition of calcium salts in the vascular walls [16,17,18,19,20]. This phenomenon is linked to arterial stiffness and endothelial dysfunction, factors that collectively contribute to the progression of atherosclerosis and increased cardiovascular risk [16,17,18,19,20]. The intricate balance of tissue mineralization is disrupted through pyrophosphate inhibition, an imbalance that can further exacerbate vascular calcification and compromise overall vascular health [6]. Moreover, the involvement of ALP in interactions with inflammatory cytokines introduces an additional layer of complexity to its role in cardiovascular pathophysiology [21]. The promotion of vascular smooth muscle cell calcification and inflammation by these interactions underscores the intertwined nature of inflammatory processes and vascular health [22]. These cascading effects can contribute to the progression of atherosclerosis, a key player in the development of cardiovascular diseases. In the context of atherosclerosis, ALP emerges as a potential participant in the destabilization of atherosclerotic plaques [23]. The structural integrity of these plaques is crucial, and any factor contributing to their destabilization can elevate the risk of adverse cardiovascular events. Therefore, ALP’s involvement in this process adds to its significance as a potential mediator of cardiovascular risk [23].

The existence of specific ALP isoforms, despite their limited clinical use hitherto, is noteworthy [24]. Human ALP, comprising four distinct isoenzymes, can be dissected using chromatography techniques [6]. Among these isoenzymes, tissue-nonspecific ALP (TNALP) takes precedence, constituting over 90% of circulating ALP and emerging as the most abundant isoenzyme in the human body [6]. TNALP, in turn, encompasses two critical isoforms: bone-specific and liver-specific TNALP, each characterized by distinct enzymatic activities and localizations within the body [6]. Circulating ALP levels comprise mostly liver and bone subfractions in nearly equal proportions, while intestinal, kidney, and placental ALP contribute the least [6,24]. Subsequently, various pathologic conditions affecting liver and bone function and metabolism, as well as physiological growth and aging, impact serum ALP levels accordingly [6,24]. In developing children, ALP levels appear 1.5 to 2.5 times that of a normal adult, while in conditions like cholestasis, Paget’s disease, bone metastases and primary tumors, osteomalacia, and rickets, ALP levels spike from moderate to high values, 10–25 times the normal limit [24]. Conversely, in achondroplasia, cretinism, or hypothyroidism, lower values are present [24]. The average age of our study participants (57 years old) coincides with the period characterized by the onset of osteoporotic changes and heightened bone turnover. Consequently, it is plausible that ALP activity at this stage of life primarily reflects bone metabolism rather than inflammatory processes that could potentially influence the development of ASCVD. However, it is important to emphasize the typically normal ALP levels observed in individuals with osteoporosis, thereby excluding this condition from the roster of potential confounding factors involved in the complex interplay between ALP and ASCVD. This intricate composition underscores the need for a comprehensive exploration of the various forms of ALP and their individual contributions to cardiovascular health [6]. Despite the complexity of the isoenzyme composition of ALP, the majority of studies have traditionally focused on investigating the association of serum circulating ALP with CVD and all-cause mortality without delving into the specific contributions of each isoenzyme. This presents an avenue for future research to unravel the distinct roles played by these isoforms in cardiovascular health, potentially revealing novel insights into the mechanisms highlighting the association of ALP with adverse cardiovascular outcomes.

Conversely, the potential therapeutic benefits of reducing ALP through pharmacological intervention in individuals with established ASCVD have been proposed. An insightful analysis conducted across three phase 2 placebo-controlled trials involving 795 patients with diabetes and recent acute coronary syndrome has shed light on the potential cardiovascular advantages of ALP reduction. Specifically, a noteworthy finding emerged, indicating that a 1 SD reduction in ALP was associated with a marked reduction in MACE (HR: 0.64; 95% CI: 0.46–0.90, *p* = 0.009) [9]. The focal agent of this investigation was apabetalone, an oral medication distinguished by its capacity to induce epigenetic modifications of gene transcription. Operating through the modification of bromodomain and extra-terminal (BET) proteins, apabetalone represents a promising avenue for targeted intervention [25]. The body of evidence, derived from both experimental models and human studies, points towards favorable effects of apabetalone on multiple facets of CVD, encompassing atherosclerosis, inflammation, and vascular calcification [9]. Furthermore, the investigation draws parallels between the effects of apabetalone and statin therapy. Statins, renowned for their established role in lipid management, have demonstrated a positive impact on coronary and aortic valve calcification [26]. This phenomenon has been attributed to the inhibition of ALP expression, as elucidated through in vitro models [26]. The implications of statin therapy extend beyond lipid regulation, encompassing potential anabolic effects on bone tissue through intricate molecular pathways [27,28]. These pathways include the reduction in bone turnover, as evidenced by a study demonstrating an inverse correlation between the reduction in serum plasma bone markers following statin therapy and bone mineral density [29]. Likewise, a recent study has investigated the impact of statin therapy on bone metabolism in postmenopausal women with hyperlipidemia [30]. It was a sub-analysis of a larger trial comparing the effects of different statins on coronary artery calcium (CAC) scores [30]. Bone attenuation was assessed using thoracic vertebrae computerized tomography scans at baseline and 12 months [30]. Interestingly, bone attenuation loss was significantly greater with pravastatin compared to atorvastatin, suggesting a potential negative impact of pravastatin on bone health [30]. This attenuation loss was comparable to that seen in an untreated general population sample [30]. Furthermore, the study found an inverse correlation between changes in LDL-C and total cholesterol with bone attenuation change, indicating a potential link between lipid levels and bone health [30]. Multivariable analysis identified race and percent change in LDL-C as independent predictors of bone attenuation change [30]. Notably, other factors like age, body mass index, and medical history did not significantly affect bone mineral density change [30]. Thus, the authors concluded that their results suggest an interaction between lipid metabolism and bone health [30]. This evidence highlights the importance of considering the effects of statins on bone metabolism in addition to their cardiovascular benefits. However, despite the intriguing associations between statin-induced ALP reduction, bone turnover, and vascular health, the current body of evidence falls short in establishing a definitive link between statin-induced ALP reduction and a subsequent decrease in ASCVD events. Consequently, a critical gap exists in our understanding of the complex interplay between statins, ALP modulation, and cardiovascular outcomes. The paucity of data underscores the imperative for further research in this field to elucidate the relationships and any potential therapeutic implications.

### Study Limitations

The retrospective nature of our study introduces challenges associated with data collection. Causality inference is constrained by the observational nature of our study, preventing definitive conclusions regarding the cause-and-effect relationship between elevated ALP levels and incident ASCVD. Furthermore, our study lacks comprehensive data on inflammatory markers, such as CRP, limiting our understanding of the role of inflammation in the identified associations. In the same context, changes in blood pressure or lipid-lowering therapies during the observational period are not discussed, and their potential influence on the reported findings must be acknowledged. Dietary habits and physical activity, recognized contributors to incident ASCVD, were not included in our study, leading to an incomplete assessment of lifestyle factors. The diversities noted across the subjects’ groups stratified by alkaline phosphatase and lipid-lowering treatment could have influenced our results. Likewise, the significant differences noted in some clinical parameters, primarily encompassing systolic and diastolic blood pressure, as well as total and LDL-C and triglyceride levels, could potentially have a significant impact from a clinical perspective. These variations may also play a crucial role in influencing the risk of atherosclerotic cardiovascular disease in the ensuing years. Moreover, this study did not perform a detailed analysis of ALP isoenzymes, preventing an understanding of their contributions to ASCVD risk. Likewise, the absence of electrophoresis data limits the ability to conduct an isoform-specific analysis of ALP, hindering insights into the distinct roles of specific ALP isoforms in ASCVD. Finally, this study population primarily comprised dyslipidemic subjects, potentially restricting the generalizability of findings to diverse demographic groups. 

While our study offers valuable insights into ALP and ASCVD associations, these limitations underscore the need for cautious interpretation. Future research addressing these limitations will enhance the applicability of findings in guiding clinical practice and interventions related to ALP and cardiovascular health.

## 5. Conclusions and Future Perspectives

The findings from the present study provide compelling evidence suggesting a potential association between elevated ALP levels and an augmented ASCVD risk. Our data align with the numerous publications highlighting the association of ALP with adverse ASCVD outcomes. However, despite the observed association, the precise role of ALP in serving as either a reliable prognostic marker or a plausible therapeutic target remains uncertain. Importantly, the relatively small sample size and the descriptive nature of our analysis should be considered when interpreting our results. These limitations constrain our ability to draw robust conclusions regarding causality and prognostic value. Further in-depth investigations are needed to delineate the intricate relationship between ALP and ASCVD risk, particularly focusing on exploring the contributions of specific ALP isoforms. A more nuanced understanding of the isoform-specific associations could elucidate the underlying mechanisms and facilitate the development of targeted interventions. All things considered, the present study serves as a steppingstone, underscoring the importance of future research endeavors to unlock the full potential of ALP as a predictive tool and potential therapeutic target in the realm of ASCVD prevention within the dyslipidemic population.

## Figures and Tables

**Table 1:** Baseline characteristics of study participants.

		Tertiles of Alkaline Phosphatase			
	Total Sample	Low	Middle	High	*p*
*N*	1178	383	398	397	
Sex (male), %	44	49	42	43	NS
Age, yrs	57 (49–65)	55 (47–64)	57 (51–66)	58 (49–66)	0.01
Follow-up, years	6 (4–9)	5 (3–8)	5 (4–8)	8 (5–12)	<0.001
Smoking, %	32	40	27	29	<0.001
Hypertension, %	61	59	59	64	NS
Diabetes, %	11	9	11	12	NS
Atherosclerotic Cardiovascular Diseaser, %	16	15	15	18	NS
Chronic kidney disease, %	10	9	10	10	NS
Systolic blood pressure, mmHg	140 (125–150)	134 (120–150)	138 (126.5–150)	140 (130–160)	<0.001
Diastolic blood pressure, mmHg	85 (80–93)	83 (78–91)	84 (78–90)	90 (80–95)	<0.001
Fasting plasma glucose, mg/dL	96 (88–106)	93 (87–103)	96 (88–106)	97 (89–109)	<0.001
Total cholesterol, mg/dL	250 (212–286)	246 (205–271)	248 (212–289)	257 (222–301)	<0.001
Triglycerides, mg/dL	129 (94–186)	121 (89–171)	130.5 (92–189.5)	137 (100–194)	<0.01
High-density lipoprotein cholesterol, mg/dL	52 (44–62)	52 (45–63)	54 (45.5–65)	50 (42–60)	<0.001
Low-density lipoprotein cholesterol, mg/dL	166.2 (131.8–195.4)	161 (125.5–187.7)	164.3 (130–193.2)	173 (141–207.6)	<0.001
Alkaline Phosphatase, IU/L	67 (54–90)	50 (44–54)	67 (63–72)	112 (90–169)	<0.001
Lipid-lowering therapy, %	23	26	24	18	<0.05
Statin Therapy, %	21	23	23	17	0.053
Moderate Intensity, %	13	13	15	11	NS
High Intensity, %	8	10	8	5	NS
Ezetimibe, %	1	1	2	1	NS
Fibrates, %	2	3	1	1	NS
Omega-3 fatty acids, %	1	2	1	1	NS
Antihypertensive therapy, %	46	44	45	48	NS
Antidiabetic therapy, %	7	8	9	8	NS

Tertiles of alkaline phosphatase were defined as the following: low: <67 U/L; middle: 67–79 U/L; high: ≥79 U/L.

**Table 2:** Association between alkaline phosphatase and incident atherosclerotic cardiovascular disease.

	Total Study Participants		
	Model 1	Model 2	Model 3
Alkaline Phosphatase (continuous variable) * Alkaline Phosphatase tertiles	8.67 (3.21–23.38), *p* <0.001	9.10 (3.22–25.75), *p* <0.001	6.99 (2.29–21.03), *p* = 0.001
Low	Reference	Reference	Reference
Middle	1.15 (0.58–2.27), *p* = 0.687	1.13 (0.57–2.25), *p* = 0.725	1.19 (0.59–2.39), *p* = 0.621
High	2.79 (1.54–5.04), *p* = 0.001	2.72 (1.49–4.98), *p* = 0.001	2.35 (1.24–4.41), *p* = 0.008

Associations are expressed as odds ratios (95% confidence intervals). Model 1: crude analysis. Model 2: adjusted for sex and age. Model 3: adjusted for sex, age, hypertension, diabetes, smoking, baseline atherosclerotic cardiovascular disease, triglycerides, high- and low-density lipoprotein cholesterol, and lipid-lowering therapy. * Alkaline phosphatase (continuous variable) was logarithmically transformed.

## Data Availability

Data is unavailable due to privacy restrictions.

## Supplementary Materials

The following supporting information can be downloaded at: https://www.mdpi.com/article/10.3390/jcdd11020060/s1, Table S1: Baseline laboratory variables across tertiles of alkaline phosphatase; Table S2: Participants’ alkaline phosphatase levels and metabolic profile stratified by lipid-lowering therapy.
