# Supplementary material for: Association of Alkaline Phosphatase with Cardiovascular Disease in Patients with Dyslipidemia: A 6-Year Retrospective Study
Source: J Cardiovasc Dev Dis. 2024 Feb 15;11(2):60. doi: 10.3390/jcdd11020060 (PMC10889667; doi:10.3390/jcdd11020060)
Supplement: Supplementary file 1 [file jcdd-11-00060-s001.zip › jcdd-2827972-supplementary.pdf]

Supplementary Material

Table S1 Baseline laboratory variables across tertiles of alkaline phosphatase.

	Tertiles of alkaline phosphatase				
	**Total Sample**	**Lower**	**Middle**	**Highest**	**p**
**Estimated glomerular filtration rate, mL/min/1.73 m^2**	80.7 (70.57-91.79)	83 (72.24-92.35)	80.79 (71.98-90.51)	78.63 (68.53-92.21)	NS
**Aspartate aminotransferase, IU/L**	21 (18-25)	21 (18.25)	22 (18-26)	21 (18-25)	NS
**Alanine aminotransferase, IU/L**	22 (17-29)	21 (16-28)	22 (17-29)	22 (17-29)	NS
**Gamma-glutamyltranspetidase, IU/L**	18 (13-28)	18 (12-25.5)	18 (13-28)	20 (14-30)	NS
**Total bilirubin, mg/dL**	0.6 (0.5-0.8)	0.7 (0.5-0.8)	0.6 (0.5-0.8)	0.6 (0.5-0.8)	NS
**Direct bilirubin mg/dL**	0.1 (0.08-0.13)	0.11 (0.09-0.14)	0.1 (0.08-0.13)	0.09 (0.07-0.12)	<0.05
**Hemoglobin, g/dL**	14 (13.2-14.9)	14.1 (13.2-15.1)	13.9 (13.1-14.9)	13.9 (13.1-14.6)	NS
**Calcium, md/dL**	9.7 (9.4-10)	9.7 (9.4-10)	9.76 (9.4-10)	9.6 (9.4-9.9)	<0.05
**Phosphate, mg/dL**	3.3 (3-3.7)	3.2 (2.9-3.6)	3.3 (3-3.8)	3.4 (2.95-3.7)	NS
**Albumin, g/dL**	-	4.4 (4.3-4.6)	4.4 (4.2-4.6)	4.5 (4.3-4.7)	NS

Tertiles of alkaline phosphatase were defined as the following: lowest: <67 U/L, middle: 67-79 U/L, highest: ≥79 U/L

Table S2 Participants' alkaline phosphatase levels and metabolic profile stratified by lipid-lowering therapy.

	**No lipid-lowering therapy**	**Lipid-lowering therapy**	**p**
**Alkaline Phosphatase, IU/L**	69 (55-92)	64 (53-82)	<0.05
**Systolic blood pressure, mmHg**	140 (126-150)	134 (123-150)	<0.05
**Diastolic blood pressure, mmHg**	86 (80-95)	80 (75-90)	<0.05
**Fasting plasma glucose, mg/dL**	95 (87-105)	99 (90-112)	<0.05
**Total cholesterol, mg/dL**	257 (228-291)	205 (179-246)	<0.05
**Triglycerides, mg/dL**	131 (94-188)	127 (90-179)	NS
**High-density lipoprotein cholesterol, mg/dL**	52 (45-64)	51 (43-60)	<0.05
**Low-density lipoprotein cholesterol, mg/dL**	173 (146-201)	124 (100-164)	<0.05
